# Warburg effect hypothesis in autism Spectrum disorders

**DOI:** 10.1186/s13041-017-0343-6

**Published:** 2018-01-04

**Authors:** Alexandre Vallée, Jean-Noël Vallée

**Affiliations:** 1 Laboratoire de Mathématiques et Applications (LMA), UMR CNRS 7348, CHU Poitiers, University of Poitiers, Poitiers, France; 2 Laboratoire de Mathématiques et Applications (LMA), UMR CNRS 7348, University of Poitiers, 11 Boulevard Marie et Pierre Curie, Poitiers, France; 3 CHU Amiens Picardie, Université Picardie Jules Verne (UPJV), Amiens, France

**Keywords:** WNT/β-catenin pathway, Aerobic glycolysis, Warburg effect, Lactate, Autism spectrum disorders, LDH-a

## Abstract

Autism spectrum disorder (ASD) is a neurodevelopmental disease which is characterized by a deficit in social interactions and communication with repetitive and restrictive behavior. In altered cells, metabolic enzymes are modified by the dysregulation of the canonical WNT/β-catenin pathway. In ASD, the canonical WNT/β-catenin pathway is upregulated. We focus this review on the hypothesis of Warburg effect stimulated by the overexpression of the canonical WNT/β-catenin pathway in ASD. Upregulation of WNT/β-catenin pathway induces aerobic glycolysis, named Warburg effect, through activation of glucose transporter (Glut), pyruvate kinase M2 (PKM2), pyruvate dehydrogenase kinase 1(PDK1), monocarboxylate lactate transporter 1 (MCT-1), lactate dehydrogenase kinase-A (LDH-A) and inactivation of pyruvate dehydrogenase complex (PDH). The aerobic glycolysis consists to a supply of a large part of glucose into lactate regardless of oxygen. Aerobic glycolysis is less efficient in terms of ATP production than oxidative phosphorylation because of the shunt of the TCA cycle. Dysregulation of energetic metabolism might promote cell deregulation and progression of ASD. Warburg effect regulation could be an attractive target for developing therapeutic interventions in ASD.

## Background

Autism spectrum disorders (ASD) is a neurodevelopmental disease which is characterized by a deficit in social interactions and communication with repetitive and restrictive behaviors [1], poor eye contact [2] and disruption of cognitive and motor development [3]. ASD is mainly diagnosed within the first three years of life. Early diagnosis is critical for better prognosis and therapeutic care [4, 5]. 10% of ASD cases are associated with a “genetic syndromic ASD” and the other cases, as “idiopathic ASD” and “primary ASD”, have no clearly known causes. Several genetic factor and environmental effects may contribute to the heterogeneity etiologic of this disease [6]. However, the etiology of ASD remains unknown.

Dysregulation of the core neurodevelopmental pathways is associated with the clinical presentation of ASD, and one of the major pathways involved in developmental cognitive disorders is the canonical WNT/β-catenin pathway [7]. Several genetic mutations observed in ASD are linked with the deregulation of the canonical WNT/β-catenin pathway by interactions between chromodomain helicase DNA binding protein 8 (CDH8) and CTNNB1 (β-catenin) [8]. Canonical WNT/β-catenin pathway has a critical role in the development of the central nervous system (CNS), and is over-expressed in ASD [7, 9, 10].

Metabolic enzymes are modified by the dysregulation of the canonical WNT/β-catenin pathway. Upregulation of WNT/β-catenin signaling leads to activation of pyruvate dehydrogenase kinase-1 (PDK-1), which decreases the activity of the pyruvate dehydrogenase complex (PDH). Upregulation of WNT/β-catenin signaling also activates monocarboxylate lactate transporter-1 (MCT-1) [11]. This do not allow the conversion of pyruvate into acetyl-coenzyme A (acetyl-CoA) in mitochondria and its entry into the tricarboxylic acid (TCA) cycle. At this stage, cytosolic pyruvate is converted into lactate for the major party. This phenomenon is called Warburg effect or aerobic glycolysis despite the availability of oxygen [12].

Mitochondrial deregulation is one of the main metabolic abnormalities observed in ASD physiopathology [13–17]. Several studies have shown a significant increase in lactate dehydrogenase kinase A (LDH-A) expression and pyruvate levels [18] with an increased lactate/pyruvate ratio [19], and elevated levels of lactate in ASD patients [20, 21].

There is some common denominator between these metabolic abnormalities, which strongly suggests the reprogramming of cellular energy metabolism with increase lactate production induced by over-expressed canonical WNT/β-catenin pathway in ASD.

We focus this review on the hypothesis of Warburg effect induced by over-expressed canonical WNT/β-catenin pathway in ASD.

### Canonical WNT/β-catenin pathway

Wingless and integration site (called WNT) pathway is a cascade of several signaling implicated in development, growth, and metabolism [22]. WNT signaling is composed by secreted lipid-modified glycoproteins [23]. WNT/β-catenin pathway is involved in numerous mechanisms such as patterning, development of synapses in the CNS [24, 25], synaptogenesis [26, 27] and the control of synaptic formation [24, 28].

Dysregulation of the canonical WNT/β-catenin pathway is observed in numerous diseases [29], such as cancers, as gliomas [30, 31] and colon cancer [32], and neurodegenerative diseases as Alzheimer’s disease [33, 34], age macular degeneration [35, 36], amyotrophic lateral sclerosis [37] and multiple sclerosis [38] (Table 1).

**Table 1:** Canonical WNT/β-catenin pathway dysregulation

WNT/β-catenin pathway	Pathologies	References
Increase	Age-macular degeneration	[35, 36]
	Aging	[113]
	Amyotrophic lateral sclerosis	[37]
	Atherosclerosis	[114]
	Cancers	[97]
	Colon cancer	[115]
	Diabetes 2	[32]
	Fibrosis	[116, 117]
	Gliomas	[30, 31]
	Huntington’s disease	[118]
	Multiple sclerosis	[34]
	Radiation-induced fibrosis	[119]
Decrease	Alzheimer’s disease	[33, 34, 120]
	Arrhythmogenic right ventricular cardiomyopathy	[121]
	Bipolar disorder	[122]
	Osteoporosis	[123]
	Parkinson’s disease	[124]

WNT family genes are 19 members which are classified as canonical and non-canonical WNT pathway. Canonical WNT ligands are seven, as WNT1, WNT2, WNT3, WNT8a, WNT8b, WNT10a and WNT10b). They are activators of the WNT/β-catenin pathway. Canonical WNT ligands are secreted by neurons and immune cells in the CNS [39]. The non-canonical WNT pathway is independent to β-catenin signaling and is separated into the planar cell planar cell polarity pathway and the WNT/Ca^2+^ pathway.

WNT extracellular ligands bind low density lipoprotein receptor-related protein 5 and 6 (LRP 5/6), Frizzled (FZD) receptors, and then disheveled (DSH), resulting in β-catenin accumulation and nuclear translocation. Thus, N-nuclear β-catenin bind T-cell factor/lymphoid enhancer factor (TCF/LEF) [40]. The complex formed TCF/LEF–nuclear β-catenin leads to the stimulation and the transcription of several WNT target genes (c-Myc, cyclin D1) [41].

The absence of binding between membrane receptors and WNT extracellular ligands characterizes the downregulation of WNT/β-catenin pathway. The β-catenin complex destruction is formed by adenomatous polyposis coli (APC), AXIN and glycogen synthase kinase-3β (GSK-3 β). This complex binds β-catenin to degrade it into the proteasome [42]. Activated GSK-3β downregulates β-catenin accumulation and its nuclear translocation [42, 43].

#### WNT/β-catenin pathway and PI3K/Akt pathway

Phosphatidylinositol 3-kinase/serine/threonine kinase (protein kinase B)/mammalian target of rapamycin (PI3K/Akt/mTOR) pathway is implicated in proliferation, growth, protein synthesis and metabolism [44–47]. WNT/β-catenin pathway, through the inhibition GSK-3β activity [48], is considered as one of the main activator of PI3K/Akt/mTOR pathway [49]. GSK-3β, a major inhibitor of the WNT ligands [50], is a specific intracellular serine-threonine kinase which regulates numerous pathophysiological pathways [51–53]. PI3K/Akt pathway decreases the activity of GSK-3β in adipocyte differentiation [54, 55]. In addition, decrease of β-catenin levels downregulates the expression of PI3K/Akt/mTOR pathway [56, 57].

#### Canonical WNT/β-catenin and PI3K/Akt pathways in ASD

Several studies have shown the major role of activated WNT/β-catenin pathway in ASD [58–60]. Numerous genetic components are correlated with ASD development such as WNT2 ligand [61], hepatocyte growth factor receptor (MET) which is a WNT target gene [62, 63], and chromo-helicase domain protein 8 (CHD8) and DYRK1A which can both modulate WNT/β-catenin pathway [64–66].

Several studies has shown a main role of numerous compounds of the WNT/β-catenin pathway in ASD, such as WNT1 [67], WNT2 [61], WNT3 [68], WNT7A [69], APC [70–72], β-catenin [8, 73], TCF4 [74, 75] and TCF7 [76].

The knockout of the gene encoding phosphatase and tensin homolog protein (PTEN), a cytoplasmic protein suppressor of WNT/β-catenin pathway, has been identified as a high-risk ASD susceptibility gene [77–80]. PTEN is also a negative regulator of PI3K/Akt pathway [81] and deletion of PTEN expression leads to stimulate proliferation and migration through the activation of mTOR activity [82]. Knockout of PTEN in Purkinje cells impairs social relation, behavior and deficits in motor learning [83, 84]. PTEN and β-catenin regulate each other leading to normal growth of the brain [85].

#### Valproate and ASD

Valproate (or Valproic acid, VPA) is an anti-convulsing agent discovered in 1963 and used for treatment of bipolar disorders or migraine [86, 87]. VPA decreases GSK-3β activity and then stimulates WNT/β-catenin pathway [88–90].

In neural stem cells of the CNS, VPA can increase WNT3a expression and β-catenin accumulation [90]. In rat models, treatment with VPA activates WNT/β-catenin pathway and inhibits GSK-3β activity, which stimulates PI3K/Akt/mTOR pathway [89, 91]. VPA increases the risk of ASD in pregnant woman during prenatal development through the stimulation of WNT/β-catenin pathway [92].

### Warburg effect

The Warburg effect (also named aerobic glycolysis) consists to a conversion of a large part of glucose into lactate regardless of oxygen [12]. Activated PDK1 phosphorylates the PDH in order to stop the conversion of pyruvate into acetyl-coA in mitochondria [93]. This conversion is proportionally diminished with a consequent reduction of acetyl-CoA entering the tricarboxylic acid (TCA) cycle. Then, cytosolic pyruvate being towards the formation of lactate which is then expelled from the cell by the upregulation of both lactate dehydrogenase A (LDH-A) and MCT-1. The higher production of lactate through this action favors anabolic production of biomass, and nucleotide synthesis [94]. However, the oxidative phosphorylation stays more efficient in terms of ATP production than aerobic glycolysis because of the shunt of the TCA cycle. PDK transcription is also regulated by insulin, glucocorticoids, thyroid hormone and fatty acids [95] which allow the metabolic flexibility [94].

#### Warburg effect activation through canonical WNT/β-catenin pathway stimulation (Fig. 1)

Several studies have shown that aerobic glycolysis is induced by overactivation of the WNT/β-catenin pathway through a direct activation of PDK1 and MCT-1 [31, 35, 96, 97]. β-catenin activation induces the expression of PI3K/Akt signaling [56, 57].

**Fig. 1:**
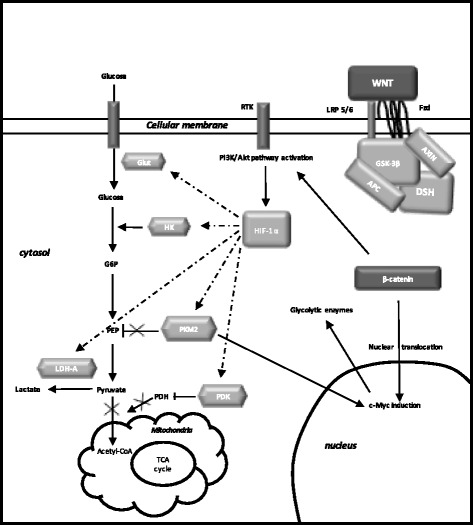
Relation between activated WNT/β-catenin pathway and Warburg effect in ASD. Mutations in ASD lead to activate the presence of WNT ligands. Then, WNT binds both Frizzled and LRP 5/6 receptors to phosphorylate the AXIN/APC/GSK-3β complex. Thus, β-catenin phosphorylation is stopped and this inhibits its degradation into the proteasome. β-catenin accumulates in the cytosol and translocates to the nucleus to bind the complex TCF/LEF co transcription factors. WNT target gene transcription is activated by nuclear β-catenin (PDK, c-Myc, cyclin D1, MCT-1). Glucose also activates the WNT signaling. MCT-1 favors lactate expulsion out of the cell. WNT/β-catenin pathway activates tyrosine kinase receptors (TKRs) activity. Activated PI3K/Akt pathway stimulates glucose metabolism. Akt-transformed cells protect against reactive oxygen species stress (ROS) by inducing HIF-1α, which suppresses glucose entry into the TCA cycle. Stimulation of HIF-1α activity activates the expression of the glycolytic enzymes (GLUT, HK, PKM2, LDH-A). Aerobic glycolysis is observed with the increase of lactate production and the decrease of mitochondrial respiration. HIF-1α induced PDK phosphorylates PDH, which resulting in cytosolic pyruvate being shunted into lactate by inducing LDH-A activation. PDK inhibits the PDH complex into the mitochondria, thus pyruvate cannot be fully converted into acetyl-CoA and enter the TCA cycle. c-Myc and cyclin D1 also stimulates LDH-A activity which converts cytosolic pyruvate into lactate. Activated PKM2 translocates to the nucleus to bind β-catenin and then to induce the expression of c-Myc

Increase rate of glucose metabolism is associated with the overactivation of PI3K/Akt pathway [98]. Activation of PI3K/Akt pathway stimulates HIF-1α (hypoxia-inducible factor 1-α) [99], which induces stimulation of glycolytic enzymes such as Glut, LDH-A, PDK1 and PKM2 [99, 100].

Glut-1 and Glut-3 are mainly important for the insulin-sensitive homeostasis of glucose transport [101]. Then, the conversion of phosphoenolpyruvate (PEP) and ADP into pyruvate is the final step in glycolysis after glucose entered the cell. The enzyme pyruvate kinase (PK) catalyzes this reaction. PK have four isoforms: PKM1, PKM2, PKL, and PKR. The dimeric form of PKM2 has low affinity with PEP [102]. Under high glucose concentration, PKM2 is translocated to the nucleus through the action of peptidyl-prolyl isomerase 1 (Pin1) [103], which reduces its activity and targets PKM2 toward lysosome-dependent degradation [104]. Nuclear PKM2 binds nuclear β-catenin and then induces c-Myc-mediated expression of glycolytic enzymes including Glut, LDH-A, PDK1, and PKM2 [105].

Activated c-Myc also activates glutaminolysis and tends to nucleotide synthesis [106] by activating HIF-1α which controls PDK1 [107]. A minor part of the pyruvate is converted into acetyl-CoA which enters the TCA cycle and become citrate for promoting protein and lipid synthesis.

#### Lactate production in ASD

Up to now, few studies have described the expression of the different glycolytic enzymes in ASD. However, several studies have shown elevated lactate levels in ASD patients [14, 18–21, 108–110]. In the same way, production of pyruvate is stimulated [20, 110] but with an increased ratio lactate-to-pyruvate [19, 20]. A recent study has observed a significant increase in LDH-A expression and pyruvate levels in ASD [18]. A recent study have shown a decrease level of pH associated with the overproduction of lactate in ASD [111]. These findings may suggest an elevation of glycolysis through the phenomenon of aerobic glycolysis in ASD since the dysregulation of this balance has been proposed as a candidate cause of ASD [112].

The canonical WNT/β-catenin pathway is upregulated in ASD, and is one of the major pathways involved in developmental cognitive disorders. In the present review, we examine accumulating evidence of the reprogramming of cellular energy metabolism induced by over-expressed canonical WNT/β-catenin pathway for a shift in energy production from mitochondrial oxidative phosphorylation to aerobic glycolysis as the alternative of ATP despite the availability of oxygen; a phenomenon called Warburg effect. Over-activation of the WNT/β-catenin pathway induces the transduction of WNT/β-catenin target genes, c-Myc and cyclin D1, and activates PI3K/Akt pathway, leading to HIF-1α stabilization. Both transcription of WNT-responsive genes and HIF-1α stabilization induce the transactivation of genes encoding aerobic glycolysis enzymes c-Myc, PDK, LDH-A, and MCT-1, which might explain the decreased glucose entry into the TCA cycle in mitochondria, and the conversion of a large part of glucose into lactate in cytosol, observed in the ASD. Dysregulation of cellular energy metabolism induced by over-expressed canonical WNT/β-catenin pathway might promote dysregulation and progression of the core neurodevelopmental pathways associated with the clinical presentation of ASD. Warburg effect regulation might be an innovative mechanism for therapeutic development in ASD, through the canonical WNT/β-catenin pathway as potential therapeutic target.

## Abbreviations

Acetyl-coA: Acetyl-coenzyme A
APC: Adenomatous polyposis coli
ASD: Autism spectrum disorders
CNS: Central nervous system
DSH: Disheveled
FZD: Frizzled
GLUT: Glucose transporter
GSK-3β: Glycogen synthase kinase-3β
HIF-1α: Hypoxia induce factor 1 alpha
LDH: Lactate dehydrogenase
LRP 5/6: Low-density lipoprotein receptor-related protein 5/6
MCT-1: Monocarboxylate lactate transporter-1
PDH: Pyruvate dehydrogenase complex
PDK: Pyruvate dehydrogenase kinase
PI3K-Akt: Phosphatidylinositol 3-kinase-protein kinase B
PK: Pyruvate kinase
ROS: Reactive oxygen species
TCA: Tricarboxylic acid
TCF/LEF: T-cell factor/lymphoid enhancer factor
VPA: Valproic acid
